# Unsupervised clustering analysis reveals distinct postoperative cortisol trajectories following pituitary adenoma resection in Cushing’s disease

**DOI:** 10.1007/s11060-026-05532-4

**Published:** 2026-04-30

**Authors:** Rushmin Khazanchi, Sachin Govind, Rishi Jain, Joshua Vignolles-Jeong, Vikas Munjal, Rahul Chaliparambil, Mark Damante, James P. Chandler, Luma Ghalib, Wenyu Huang, Stephen T. Magill, Daniel M. Prevedello

**Affiliations:** 1 Feinberg School of Medicine, Northwestern University, 676 N. St. Clair Street, Suite 2210, Chicago, IL 60611 USA; 2 The Ohio State University College of Medicine, Columbus, OH USA; 3 Lou & Jean Malnati Brain Tumor Institute, Northwestern University, Chicago, IL USA; 4 Department of Neurological Surgery, Northwestern University, Chicago, IL USA; 5 Department of Neurosurgery, Wexner Medical Center, The Ohio State University, Columbus, OH USA

**Keywords:** Cushing’s Disease, Pituitary adenoma, Cortisol, Transsphenoidal surgery, Machine learning, Clustering

## Abstract

**Purpose:**

Cushing’s disease (CD), caused by ACTH-secreting pituitary adenomas, results in hypercortisolism. Transsphenoidal surgery (TSS) is the first-line treatment. Early postoperative cortisol trends are frequently monitored, though standard interpretation is lacking. We sought to determine whether early postoperative cortisol trajectories are associated with biochemical remission at 1 year using unsupervised machine learning.

**Methods:**

We retrospectively reviewed 94 adult patients with CD who underwent TSS between 2011 and 2021 at two academic centers. Serum cortisol was measured every 6 h, and the resulting time series was clustered with the goal of predicting the primary outcome of biochemical remission at 1-year follow up.

**Results:**

Of 94 patients, 77 (82%) achieved biochemical remission. There were no significant differences in demographic or tumor characteristics between remission and non-remission groups. The nadir cortisol value for the remission group was lower than the non-remission group (4.8 [5.5] versus 19.3 [13.7]) though this difference was also not statistically significant (*p* = 0.1022). Clustering analysis identified three optimal clusters for remission subgroup. Statistically significant differences in cortisol trends were observed from 6 h to 30 h (p < = 0.0002). At 6 h, average cortisol values by cluster ranged from 25.51 to 61.17 mcg/dL. Two of the three clusters demonstrated an initial decrease in cortisol values, while one cluster did not immediately decrease. No significant differences in clinical, tumor, or treatment variables were observed between clusters.

**Conclusion:**

Postoperative cortisol trajectories show distinct early patterns but are not independently predictive of 1-year biochemical remission following TSS for CD.

**Supplementary Information:**

The online version contains supplementary material available at 10.1007/s11060-026-05532-4.

## Background

Cushing’s disease (CD) is an endocrine disorder caused by adrenocorticotropic hormone (ACTH)–secreting pituitary adenomas, leading to excess cortisol production from the adrenal glands. This hypercortisolism results in various clinical manifestations, including generalized weakness, hypertension, diabetes mellitus, menstrual irregularities, obesity, hirsutism, and psychiatric disturbances [1]. The first-line treatment for CD involves transsphenoidal surgery to remove the pituitary tumor responsible for the aberrant hormone secretion [2]. The primary treatment goals are the normalization of cortisol secretion, reversal of hypercortisolism symptoms, and sustained remission without recurrence. Recent data indicate that pituitary surgery achieves remission in approximately 78% of patients, with a relapse rate of around 13% over a 10-year period [3].

In the immediate postoperative period, monitoring cortisol levels is crucial for predicting remission and informing post-operative steroid administration and weaning [4–8]. Despite extensive research, preoperative clinical variables such as age, sex, disease duration, and symptom severity have not consistently identified patients at higher risk for recurrence. However, factors such as large tumor size (macroadenomas), invasive tumor behavior, and aggressive histological features are associated with increased recurrence rates [9–12].

In 2018, Mayberg et al. examined postoperative cortisol trends in patients undergoing transsphenoidal pituitary adenoma resection. The study found that patients achieving biochemical remission within six months had significantly lower serum cortisol levels at every time point within the first 72 h postoperatively and ranged from 2 to 5 ug/dL [5].

While several studies have examined factors to try and predict biochemical remission, none have incorporated temporal, serially monitored postoperative cortisol trends as input features. Variability in cortisol trajectories and their implications for prognosis remain poorly understood but may stand to challenge current paradigms that emphasize standalone postoperative values. To address these gaps, we employ k-POD clustering, an unsupervised clustering method, to better analyze temporal trends and identify patterns in recovery and remission that may deviate from clinical expectation. While clustering techniques have been used across several clinical scenarios to identify meaningful phenotypes and associated recovery profiles, they have not been used with serial cortisol levels in Cushing’s Disease patients in the post-operative period undergoing transsphenoidal surgery (TSS). This study aims to leverage machine learning to explore the predictive value of cortisol trajectories compared to demographic and tumor characteristics, understand the impact of cortisol monitoring duration on prediction accuracy, and identify key variables that influence the prediction of postoperative disease remission.

## Methods

### Study design and participants

We retrospectively reviewed patients undergoing pituitary surgery for Cushing’s Disease at two tertiary academic medical centers between 2011 and 2022. Approval for this research was granted by the hospital’s institutional review board prior to retrieval of patient clinical and radiographic data. All patients were > 18 years of age and had undergone pituitary adenoma resection with a transsphenoidal or endoscopic-endonasal approach. Exclusion criteria included: (1) lack of adequate 1-year postoperative follow-up (2) indeterminate biochemical cure status (3) history of pituitary or endocrine axis surgical intervention prior to the index surgery (4) any endocrine axis surgery within the follow-up period.

We retrospectively collected all data via chart review of laboratory studies and physician notes. Key variables of interest spanned patient demographic, tumor characteristics, and serially monitored postoperative cortisol values in 6 h intervals, with data up to 96 h post-operatively. To ensure robustness, we discarded any cortisol values after replacement steroids were administered. Biochemical remission was determined through review of clinic notes and relevant labs at 1 year after surgery. If a patient was mentioned to have biochemical cure but then was noted to have recurrence of tumor within the 1-year follow-up period, they were marked as not having achieved biochemical cure. Initial postoperative cortisol was measured at the 6-hour timepoint, and initial rate of cortisol decrease was measured between the 6 h and 18 h postoperative timepoints.

### Statistical analysis

We analyzed all independent variables (age, gender, resection approach, tumor volume, etc.) for associations with the dependent variable (biochemical cure status at 1 year follow up). We next utilized the k-POD algorithm, a novel k-means clustering implementation that is robust to missing data and variable follow-up durations [13], to separately cluster the cure and non-cure patients. To determine the optimal number of clusters (K), we used the elbow method via visual inspection of the fit integrity function for the cluster results, using the standard cluster equations from the original k-POD paper below, which measures how close data points in the same cluster are to each other (within cluster sum-of-squares, WCSS) relative to the total variation in the data from the overall mean (total sum-of-squares, TSS):

$${\rm{TSS}} = \mathop \sum \limits_{i = 1}^n {\left\| {{x_i} - \mu } \right\|^2}$$

$${\rm{WCSS}} = \mathop \sum \limits_{k = 1}^K \mathop \sum \limits_{{x_i} \in {C_k}} \left\| {{x_i} - {\mu _k}^2} \right\|$$

$${\rm{fit}} = 1 - {{{\rm{WCSS}}} \over {{\rm{TSS}}}}$$

Once clusters were defined, we compared the average cortisol value at each timepoint across clusters, as well as the differences between key demographic and tumor variables of interest across clusters. We employed one-way ANOVA (continuous variables across more than two groups), unpaired t-tests (continuous variables across exactly two groups), and Chi-square tests (categorical variables) as appropriate using standard statistical methodologies. Our sample size varied across timepoints due to different amounts of missing data. To balance cluster integrity with missing data, we first clustered patients across all 96 h of follow-up, and then re-clustered our patients based on the continuous period where statistically significant separation was achieved.

All statistical tests were corrected through the Benjamin Hochberg method for 58 statistical tests, with all p-values compared to α = = 0.0172 (Supplemental Figure 2). Statistical analysis was performed using scripts written in Python (version 3.12.9) using the pandas, numpy, scipy, kPod, and sklearn packages. Data visualization was performed using the matplotlib package.

## Results

We included 94 patients in our analysis, 77 (82%) of whom achieved biochemical remission within one year (Table 1). There was no significant difference in demographic or tumor characteristics between patients that achieved remission and patients that did not.

**Table 1:** Overall Cohort – comparison of patients with and without cure at 1 year, *n* = 94

Variable^a, b^	Remission within 1y	No Remission within 1y	All patients	*p*-value
n	77	17	94	-
**Patient variables**				
Proportion male	32 (41.6%)	8 (47.1%)	40 (42.6%)	0.8854
Age at surgery (y)	45.1 ± 15.2	43.1 ± 13.3	44.7 ± 14.9	0.5998
Length of stay (d)	3.2 ± 1.5	3.4 ± 2.0	3.3 ± 1.6	0.7261
Initial cortisol descent velocity (mcg/dL/h)	1.9 ± 1.3	3.0 ± 2.4	2.1 ± 1.5	0.3687
Initial cortisol (mcg/dL)	35.8 ± 21.7	54.0 ± 25.2	38.6 ± 23.2	0.1054
Nadir cortisol (mcg/dL)	4.8 ± 5.5	19.3 ± 13.7	6.4 ± 8.2	0.1022
**Tumor variables**				
Mean Ki67 index	0.1 ± 0.1	0.0 ± 0.0	0.1 ± 0.1	0.5097
Mean Max tumor diameter (cm)	1.0 ± 0.7	1.1 ± 0.8	1.0 ± 0.7	0.4602
Mean Tumor volume (cm³)	1.1 ± 2.4	1.7 ± 2.9	1.2 ± 2.5	0.4401
Mean Knosp grade	1.2 ± 1.3	1.7 ± 1.5	1.3 ± 1.3	0.2564
% Macroadenomas	22 (28.6%)	8 (47.1%)	30 (31.9%)	0.233
% with Apoplexy	5 (6.5%)	3 (17.6%)	8 (8.5%)	0.2146
% with Crooke’s hyaline changes	9 (11.7%)	1 (5.9%)	10 (10.6%)	0.7886
**Treatment variables**				
% with piecemeal resection approach	36 (46.8%)	7 (41.2%)	43 (45.7%)	0.8817
% with intraoperative CSF leak	10 (13.0%)	2 (11.8%)	12 (12.8%)	1.0000
% with postoperative complications	11 (14.3%)	3 (17.6%)	14 (14.9%)	1.0000
% with postoperative CSF leak	2 (2.6%)	2 (11.8%)	4 (4.3%)	0.3025

^a^ Counts and percentages are reported for discrete variables, and were compared using a χ^2^ analysis

^b^ Mean and standard deviation are reported for continuous variables, and were compared with unpaired t-tests

*Significance was defined after multiple hypothesis testing correction through the Bonferroni method, indicated with an asterisk

**Fig. 1:**
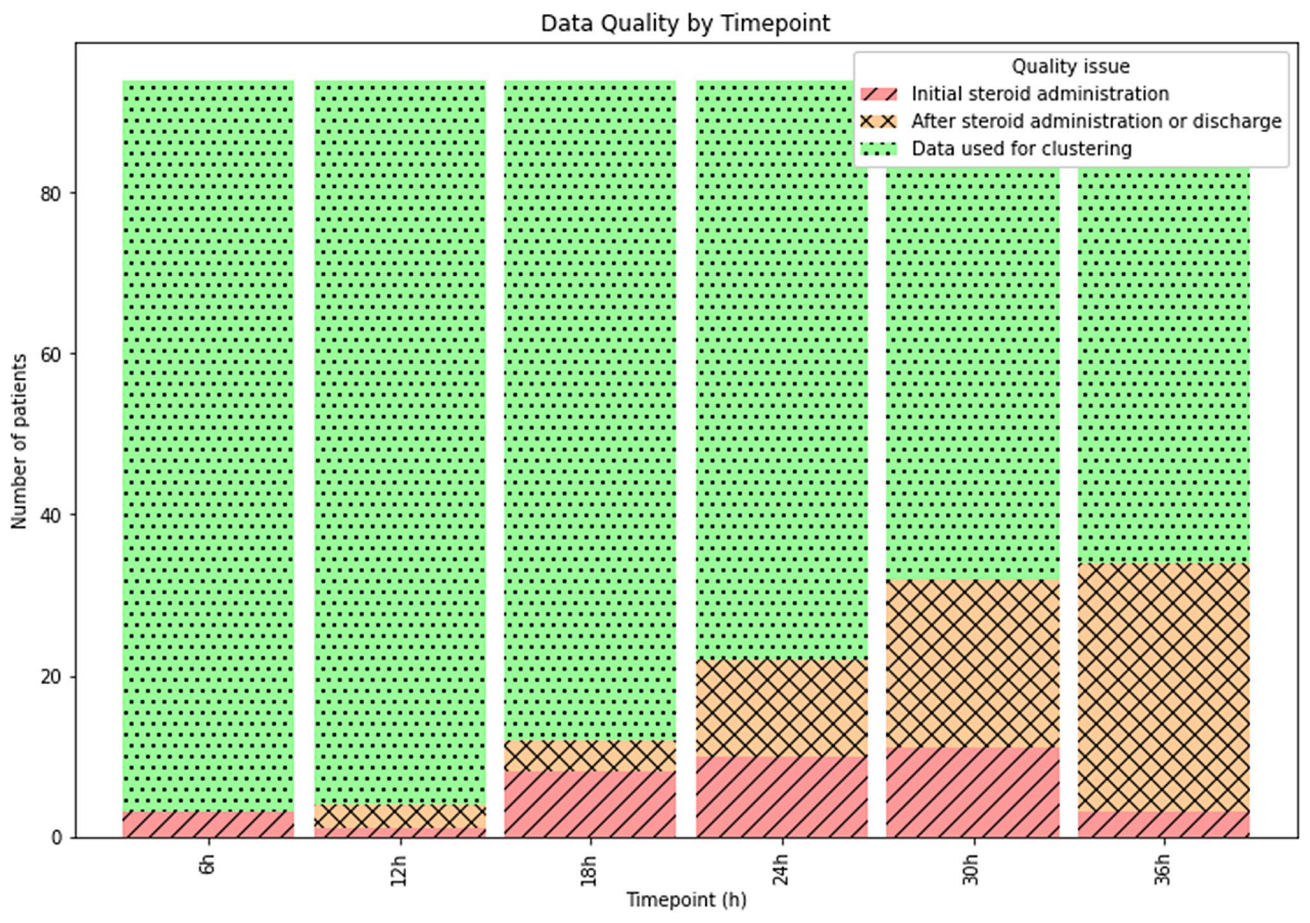
Cumulative data quality for the cohort’s cortisol trend values, assessed in the 0–36 h postoperative period. Patients received steroids at varying timepoints (red) throughout their postoperative admission; cortisol trend values after this do not hold biological significance (orange) and were nullified

The quantity of data used for clustering steadily decreased with each consecutive timepoint, as more patients either had missing data at later timepoints or had steroid replacement therapy initiated (Fig. 1). For the cure patients, clustering was performed for the first 36 h postoperatively based on curve separation (Supplemental Table 1).

**Fig. 2:**
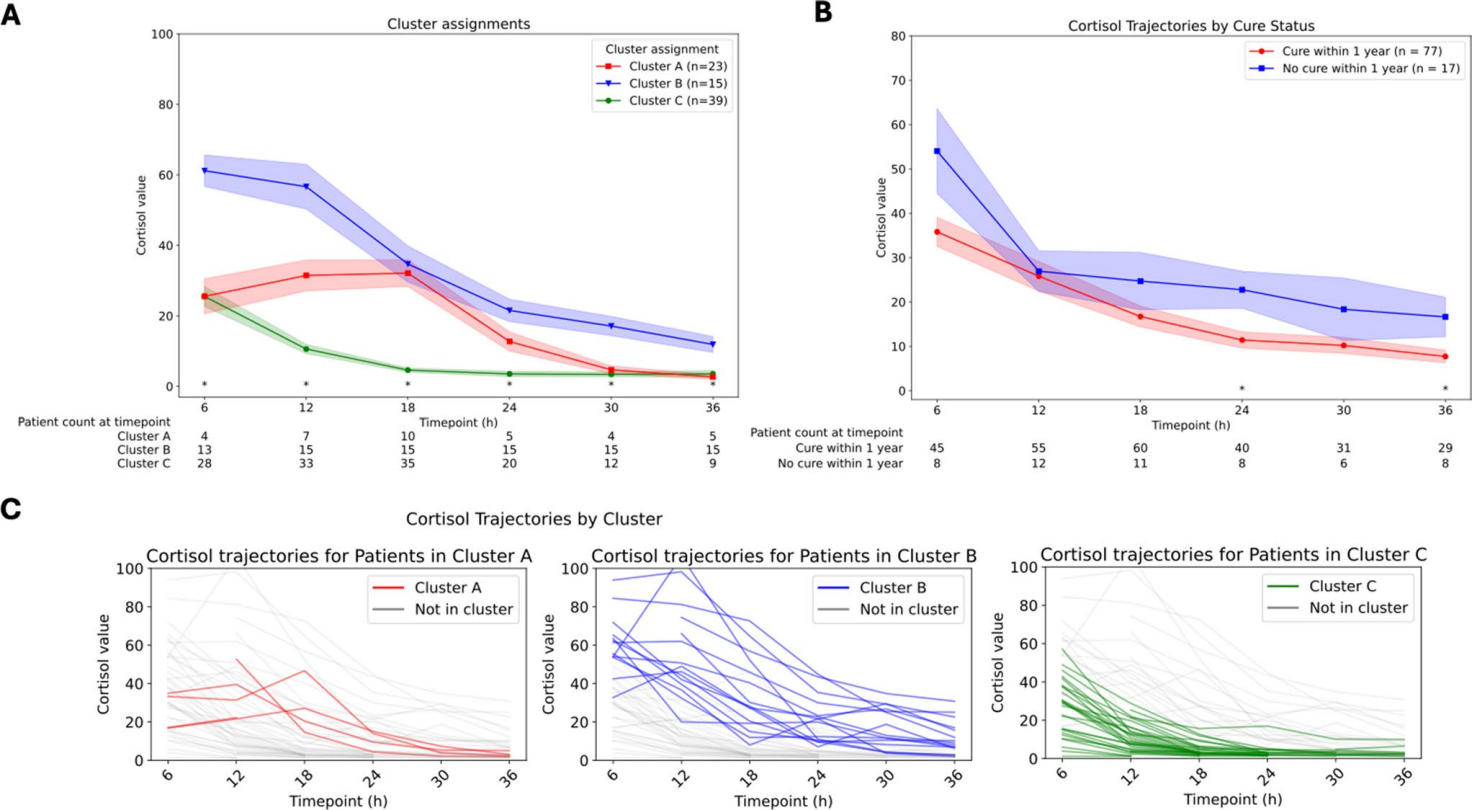
k-POD clustering results. **(A)** Cortisol trajectory for non-remission patients compared to all cure patients **(B)** Clustered cortisol trajectories for remission patients. **(C)** Individual cortisol trajectories within each cluster subgroup for patients in remission. All cortisol values are in units of mcg/dL. Patients with single timepoints of cortisol values contributed to the overall clusters in Panel B but are not shown in Panel C

Clustering failed to converge for the non-cure patients due to total sample size. Accordingly, they were treated as one group and tracked for 36 h for consistency with the cure group (Fig. 2A).

Compared to the cure patients overall, the non-cure group had higher postoperative cortisol values at all timepoints, though this did not achieve statistical significance (Table 2). The nadir cortisol value for the cure group was lower than the non-cure group (4.8 [5.5] versus 19.3 [13.7]) though this difference was also not statistically significant (*p* = 0.1022).

**Table 2:** Timepoint characteristics for cure and non-cure patients, *n* = 94

	Remission	No Remission	All patients	
Timepoint^a^Mean (SD)				
6 h	35.8 (21.7)	54.0 (25.2)	38.6 (23.2)	0.1054
12 h	25.8 (24.5)	26.9 (15.2)	26.0 (23.2)	0.8430
18 h	16.7 (18.1)	24.7 (20.4)	18.0 (18.7)	0.2669
24 h	11.4 (11.5)	22.8 (11.0)	13.3 (12.2)	0.0322
30 h	10.2 (9.9)	18.4 (15.8)	11.5 (11.5)	0.3088
36 h	7.7 (7.6)	16.6 (11.8)	9.6 (9.4)	0.0907

^a^ Mean and standard deviation are reported for continuous variables, and were compared with unpaired t-tests

*Significance was defined after multiple hypothesis testing correction through the Bonferroni method, indicated with an asterisk

Clustering analysis revealed that the optimal cluster count (K) for the cure patients was three (Fig A1), labeled as Cluster A (23 patients), Cluster B (15 patients) and Cluster C (39 patients). (Fig. 2B). Statistically significant curve separation was achieved at the 6–30 h timepoints across clusters (all p < = 0.0002). Cortisol values for each timepoint by cluster are listed in Table 3. Cluster B (Fig. 2C) had relatively higher initial cortisol values at 6 h (61.2 [15.4] mcg/dL) compared to Cluster A and C (25.5 [8.6] mcg/dL and 25.5 [14.7] mcg/dL, respectively, *p* < 0.0001). Cluster B also had a relatively higher nadir cortisol value (10.1 [7.1] mcg/dL) compared to Cluster A and C(3.5 [1.2] mcg/dL and 2.3 [1.7] mcg/dL, respectively, *p* < 0.0001). Cluster A is the only cluster where the cortisol values did not immediately descend (initial cortisol decent velocity of -0.3 [1.0] mcg/dL/h), though this was not statistically different from the other clusters (*p* = 0.0063).

**Table 3:** Timepoint characteristics for clustered cure patients for 36 h postoperatively, *n* = 77

	Cluster A	Cluster B	Cluster C	*p*-value
Timepoint^a^Mean (SD)				
6 h	25.5 (8.6)	61.2 (15.4)	25.5 (14.7)	< 0.0001*
12 h	31.5 (10.7)	56.6 (23.7)	10.6 (7.3)	< 0.0001*
18 h	32.1 (11.4)	34.7 (19.2)	4.6 (3.5)	< 0.0001*
24 h	12.7 (5.4)	21.5 (11.9)	3.5 (3.3)	< 0.0001*
30 h	4.7 (1.9)	17.1 (10.2)	3.4 (2.3)	0.0002*
36 h	2.7 (1.4)	11.9 (8.4)	3.6 (2.7)	0.0061*

^a^ Mean and standard deviation are reported for continuous variables, and were compared with unpaired t-tests

*Significance was defined after multiple hypothesis testing correction through the Bonferroni method, indicated with an asterisk

Individual patient curves appear to follow the overall cluster trajectory (Fig. 2C). There was no significant difference across clusters in patient, tumor, or treatment variables (Table 4).

**Table 4:** Post-hoc analysis of patient, tumor, and treatment characteristics across clusters, *n* = 77

	Cluster A	Cluster B	Cluster C	All	*p*-value
**Variable** ^**a, b**^					
**n**	23	15	39	77	-
**Patient variables**					
Proportion male	65.2%	46.7%	25.6%	41.6%	0.0085*
Mean Age at surgery (y)	46.7 ± 15.3	54.2 ± 10.8	40.6 ± 14.8	45.1 ± 15.2	0.0094*
Mean Length of stay (d)	3.7 ± 2.3	3.5 ± 0.6	2.8 ± 1.1	3.2 ± 1.5	0.0451
Initial Cortisol Descent velocity (mcg/dL/h)	-0.3 ± 1.0	2.3 ± 1.4	2.0 ± 1.0	1.9 ± 1.3	0.0063*
Initial Cortisol (mcg/dL)	25.5 ± 8.6	61.2 ± 15.4	25.5 ± 14.7	35.8 ± 21.7	< 0.0001*
Nadir Cortisol (mcg/dL)	3.5 ± 1.2	10.1 ± 7.1	2.3 ± 1.7	4.8 ± 5.5	< 0.0001*
**Tumor variables**					
Mean Ki67 index	0.0 ± 0.0	0.1 ± 0.1	0.1 ± 0.2	0.1 ± 0.1	0.5335
Mean Max tumor diameter (cm)	1.1 ± 0.8	0.9 ± 0.6	0.9 ± 0.7	1.0 ± 0.7	0.6106
Mean Tumor volume (cm³)	2.1 ± 3.6	0.6 ± 1.0	0.7 ± 1.6	1.1 ± 2.4	0.0533
Mean Knosp grade	1.7 ± 1.4	1.1 ± 1.3	1.0 ± 1.0	1.2 ± 1.3	0.0746
% Macroadenomas	43.5%	26.7%	20.5%	28.6%	0.1517
% Apoplexy	13.0%	0.0%	5.1%	6.5%	0.2483
% Crooke’s Hyaline changes	30.4%	0.0%	5.1%	11.7%	0.0033
**Treatment variables**					
% Piecemeal resection approach	30.4%	73.3%	46.2%	46.8%	0.0347
% Intraoperative CSF leak	17.4%	0.0%	15.4%	13.0%	0.2427
% Postoperative complications	17.4%	13.3%	12.8%	14.3%	0.8778
% Postoperative CSF leak	4.3%	0.0%	2.6%	2.6%	0.7122

^a^ Counts and percentages are reported for discrete variables, and were compared using a χ^2^ analysis

^b^ Mean and standard deviation are reported for continuous variables, and were compared with unpaired t-tests

*Significance was defined after multiple hypothesis testing correction through the Bonferroni method, indicated with an asterisk

## Discussion

Our analysis reveals three separate early postoperative cortisol trajectories following transsphenoidal surgery (TSS) for Cushing’s Disease for patients achieving biochemical remission within 1 year, namely: Cluster C - low initial post-operative cortisol, rapid fall (low/rapid) Cluster A – low initial post-operative cortisol, slow fall (low/slow); Cluster B - high initial post-operative cortisol, moderate fall (high/moderate). Our analysis highlights that the initial 6-hour cortisol value, the nadir cortisol value, and the cortisol descent velocity show statistically significant variation across the derived clusters. We also demonstrate that Cluster C had the lowest proportion of male patients as well as the youngest patients, indicating that demographic factors may have some influence on cortisol trajectory. In our cohort, even patients who did not exhibit classically expected rapid cortisol declines postoperatively, achieved biochemical remission. Our results suggest that various patterns of postoperative cortisol dynamics can result in remission and no single trajectory can be ascribed to optimal or suboptimal prognosis at largec.

Prior studies have underscored the importance of absolute cortisol levels in the postoperative period for predicting surgical success. In addition to Mayberg et al.’s findings of an ideal postoperative cortisol nadir below 2–5 µg/dL within 72 h of TSS for predicting remission [5], groups have noted that patients with persistently normal or elevated cortisol are at a higher risk of surgical failure and should receive follow up treatment [14, 15]. These findings would suggest that patients within the high/slow cluster would have demonstrably worse remission likelihoods than high/faster crashers; however, our results highlight that remission can occur even without a significant drop in cortisol levels. Such “delayed remission” has been reported; Valassi and colleagues reported that approximately 6% of Cushing’s Disease patients within their cohort displayed late cortisol decline (average of 38 days postoperatively), and although their recurrence rates were higher, they achieved remission without additional interim intervention [14]. It is plausible that our high/moderate or low/slow cluster captures such patients, emphasizing that immediate postoperative cortisol thresholds cannot be held as an absolute and definitive standard for patients. Patients who fail to acutely “crash” may still achieve meaningful delayed remission that can be captured by clinical monitoring.

Several groups have assessed clinical variables beyond postoperative cortisol trajectories as potential prognosticators. Large tumors or those presenting with greater degrees of invasion within the cavernous sinus or other structured have been associated with lower remission rates than more contained adenomas [16, 17]. Although piecemeal resections—more commonly practiced for larger and more complex lesions—were observed more frequently in clusters with slower cortisol declines, the relationship with cluster or cure rate did not achieve significance but did trend in this direction. This observation may still reflect a clinically meaningful observation, as patients with tumors requiring piecemeal resection may demonstrate slower postoperative cortisol normalization, even if cure is still achieved. Similarly, larger tumor volumes were more frequent in the slow descent cluster, although this analysis is limited by our sample size. Larger cohorts are needed to confirm this association and gauge whether the effect holds.

Patient hormone profiles and patient characteristics have been cited as potential remission predictors, including baseline ACTH, age, sex, and preoperative cortisol [16]. Though we found relationships with age and sex, our overall nonsignificant findings align with literature precedent showing inconsistent correlations between these demographic and baseline variables and treatment success [9–12]. Several confounders may make these characteristics relevant on a case-by-case basis, including the presence of comorbidities that may render patients immunocompromised or physiologically weakened [18]. Kuritsyna et al. found that patients with preoperative cortisol suppression greater than 74% in high-dose dexamethasone testing, tumor size greater than 3 millimeters on MRI (likely for visibility), and no evidence of invasiveness into the cavernous sinus, were more likely to achieve remission [19].

k-POD clustering enabled the inclusion of patients with incomplete data on cortisol trajectories to uncover latent trajectories that more straightforward regression and clustering models miss [13]. K-means clustering is commonly used to group disease phenotypes and gauge meaningful differences in outcomes, including previously in cohorts of Cushing’s Disease patients for integrated analysis of several patient outcomes [20–22]; however, it requires separate imputation and therefore is an imperfect and unrealistic fit for many real-world, large-cohort studies. Our k-POD clustering demonstrated that the concept, showing remission is possible across a spectrum of postoperative cortisol trajectories, and highlights that those who deviate from expected course of remission should not be immediately categorized as treatment “failures”. Future studies should examine the implications of these clusters over longer observations windows and in the context of other treatment characteristics, such as ACTH levels, molecular markers, and radiographic features, enabling multimodal grouping for deeper prognosis.

There are several limitations in this study. Our sample size is moderate. Limited statistical power may explain nonsignificant results, particularly regarding demographic characteristics, and cannot reliably be generalized. Second, although k-POD clustering resolved several challenges inherent to missing data values, slight heterogeneity in cortisol measurement timing may confound our findings. Similarly, given that biochemical remission was retrospectively reviewed from clinic notes, there is likely variation in the precise criteria used across institutions and endocrinologists. Prospective studies accounting for these variabilities and adopting a standardized protocol would provide more precise results and greater confidence in clustering. Third, we did not include postoperative MRI results as part of our variable set. The reason for this is that we were focused on the immediate postoperative period, during which MRI is not sensitive enough to capture residual versus expected surgically induced changes.

## Conclusions

In conclusion, we identify three unique post-operative cortisol trajectories following TSS for Cushing’s disease. All trajectories achieved biochemical remission at one year follow-up. Further validation is required to understand the clinical utility of these trajectories.

## Supplementary Information

Below is the link to the electronic supplementary material.

Supplementary Material 1

Supplementary Material 2

## Data Availability

The datasets generated during and/or analyzed during the current study are available from the corresponding author on reasonable request. The code used to generate the analysis can also be provided upon request.

## Declarations

### Presentation

A preliminary version of this paper was presented in a poster session at the Congress of Neurological Surgeons 2024 Annual Meeting and the North American Skull Base Society 2025 Annual Meeting.
