# Supplementary material for: Unsupervised clustering analysis reveals distinct postoperative cortisol trajectories following pituitary adenoma resection in Cushing’s disease
Source: J Neurooncol. 2026 Apr 30;177(3):132. doi: 10.1007/s11060-026-05532-4 (PMC13128764; doi:10.1007/s11060-026-05532-4)
Supplement: Supplementary file 2 — Supplementary Material 2 [file 11060_2026_5532_MOESM2_ESM.docx]

**Supplementary Table 1.** Timepoint analysis for cure patients across 96 hours, n = 77

|  | **Cluster A** | **Cluster B** | **Cluster C** | **p-value** |
| --- | --- | --- | --- | --- |
| **Timepoint (h)**  *Mean (SD)* |  |  |  |  |
| 6h | 20.6 ± 11.3 | 49.9 ± 12.2 | 73.4 ± 16.3 | <0.0001* |
| 12h | 10.8 ± 7.8 | 33.4 ± 12.0 | 72.1 ± 15.1 | <0.0001* |
| 18h | 9.5 ± 12.8 | 18.0 ± 10.4 | 51.8 ± 14.5 | <0.0001* |
| 24h | 4.4 ± 4.9 | 11.2 ± 6.5 | 33.1 ± 9.1 | <0.0001* |
| 30h | 2.9 ± 1.8 | 7.7 ± 4.6 | 28.5 ± 3.3 | <0.0001* |
| 36h | 2.1 ± 0.8 | 5.8 ± 2.9 | 20.5 ± 6.3 | <0.0001* |
| 42h | 9.1 ± 9.7 | 14.2 ± 9.0 | 22.4 ± 6.3 | 0.0761 |
| 48h | 1.2 ± 0.7 | 9.4 ± 4.0 | 19.5 ± 11.6 | 0.0261 |
| 54h | 1.5 ± 0.0 | 7.6 ± 2.4 | 23.9 ± 9.3 | 0.0012* |
| 60h | 2.8 ± 0.0 | 8.1 ± 4.1 | 18.5 ± 10.7 | 0.0547 |
| 66h | 16.8 ± 5.5 | 9.7 ± 6.7 | 23.6 ± 10.3 | 0.0533 |
| 72h | - | 7.2 ± 3.7 | 23.8 ± 6.0 | 0.0014* |
| 78h | - | 6.7 ± 3.9 | 13.6 ± 8.3 | 0.3178 |
| 84h | - | 9.0 ± 4.9 | 11.5 ± 7.0 | 0.8002 |
| 90h | 10.1 ± 0.0 | 4.5 ± 1.1 | 14.0 ± 0.6 | 0.0325 |
| 96h | - | 4.1 ± 0.0 | 9.5 ± 1.6 | 0.2997 |

Means and standard deviations are reported and were compared with one-way ANOVA. Significance was defined after multiple hypothesis testing correction through the Bonferroni method, indicated with an asterisk (*)


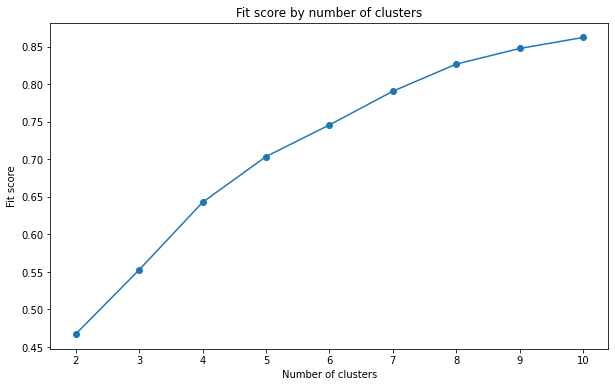


**Supplementary Figure 1.** Elbow method clustering analysis for remission patients for first 36 postoperative hours, n = 77


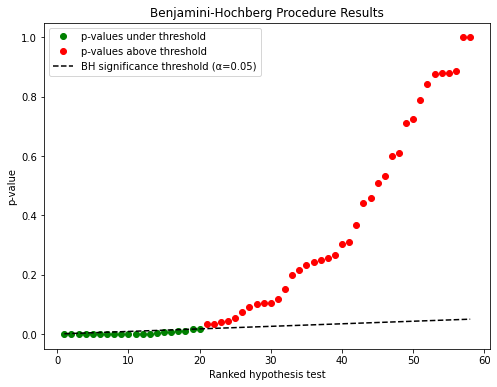


Supplementary Figure 2. Global Benjamin Hochberg correction for multiple hypothesis testing, n = 58
